# The Risk of Catastrophic Surgical Expenditure Within a Community-Based Primary and Preventive Care Program at a Florida Medical School: A Modeling Study

**DOI:** 10.7759/cureus.93545

**Published:** 2025-09-30

**Authors:** Gregory W Schneider, Jamie Fairclough, Prasad Bhoite, Anuj Ojha, Matthew T Hey, Shahab Shaffiey, Mackenzie Mayhew, Alexa Denton, Anna T LaTray, Rupa Seetharamaiah

**Affiliations:** 1 Department of Humanities, Health, and Society, Florida International University, Herbert Wertheim College of Medicine, Miami, USA; 2 Department of Data Science and Analytics, Roseman University College of Medicine, Las Vegas, USA; 3 College of Engineering, The University of Toledo, Toledo, USA; 4 Department of Surgery, Brigham and Women’s Hospital, Boston, USA; 5 Department of Pediatric Surgery, Nicklaus Children’s Hospital, Miami, USA; 6 Department of Surgery, University of Virginia School of Medicine, Charlottesville, USA; 7 Department of Surgery, Yale University, New Haven, USA; 8 Department of Medicine, Florida International University, Herbert Wertheim College of Medicine, Miami, USA; 9 Department of Surgery, Baptist Hospital of Miami, Miami, USA

**Keywords:** area deprivation index (adi), catastrophic surgical expenditure, prevention, primary care, public health, social deprivation, surgery

## Abstract

Introduction

Catastrophic surgical expenditure (CSE) poses significant financial risks globally. This modeling study investigates the risk of CSE among underserved households enrolled in a primary and preventive care program at a US community-based medical school.

Materials and methods

Using World Health Organization methodology, the analysis estimates the risk of these households suffering a CSE for an emergency cholecystectomy, adjusting for varying rates of insurance coverage. A place-based indicator of social deprivation - the Area Deprivation Index (ADI) score - was evaluated for correlation with CSE risk.

Results

Findings reveal that significant percentages of households face CSE risk, ranging from 7.7% to 88.92%, depending on insurance status and payment burden assumed. Importantly, ADI scores show a significant correlation with CSE risk. Higher ADI scores correlated with increased CSE risk, particularly for uninsured households.

Discussion

The study underscores the critical need for health insurance expansion and interventions to mitigate CSE risks, especially in low-income communities. Additionally, it proposes the use of place-based indicators like ADI to aid in identifying households at risk for CSE and to inform targeted interventions and policy discussions. Despite limitations, the study provides valuable insights into addressing financial vulnerability in healthcare and highlights avenues for further research and intervention.

Conclusion

Neighborhood-based modeling demonstrates the financial fragility of underserved communities and supports using place-based indicators like ADI in health policy, screening, and program design to reduce surgical financial catastrophe.

## Introduction

Risks of catastrophic surgical expenditure worldwide and in the United States

Worldwide, experts estimate that between 11% and 33% of the burden of disease can be treated surgically. Clinical scenarios related to obstetrics, urgent abdominal conditions, cancer, congenital anomalies, and injury are most commonly involved [1-4]. Access to affordable surgery and to healthcare in general, however, remains a global challenge. Catastrophic health expenditures (CHEs) are medical expenditures that threaten a household's financial ability to meet its subsistence needs. Catastrophic surgical expenditure (CSE) refers specifically to a CHE for surgical procedures [5]. It is estimated that 81.3 million people worldwide face financial catastrophe due to direct and indirect costs associated with surgical care. Moreover, up to 4% of individuals within the poorest quintile in high-income countries - or roughly 0.8% of the total population - are at risk of CSE [5]. Given the 2024 US population of more than 340 million, approximately 2.7 million Americans would be at risk.

In the United States, seven out of 10 uninsured trauma patients, aged 18-64, face a high risk of CSE. The greatest risk was found in the poorest communities and among the most severely injured [6]. Separate analyses led by investigators at the University of North Carolina School of Medicine, published in 2021 in Health Affairs, suggest that up to 99% of uninsured Americans facing an unexpected surgery - either because of trauma, appendicitis, cholecystitis, ovarian torsion or ectopic pregnancy, or coronary ischemia - suffer potentially catastrophic charges. The investigators noted that uninsured Americans were much more likely to present to an emergency room for surgery and to do so on a weekend, and the most common emergent procedure in the study was a cholecystectomy. The median cost of emergent surgeries in the study was $51,255, much more than the median household income at the time, of $43,463, and thus highly likely to put an uninsured patient at risk for bankruptcy [7].

As expected, insurance coverage mitigates the risk, especially in states that have expanded Medicaid and among low-income Americans who have purchased Affordable Care Act marketplace policies. Such coverage lowers the risk of CSE by approximately 35%. Insurance coverage lowers CSE risk by a variety of mechanisms. Insured individuals are more likely to present earlier for treatment, less likely to present in an emergency room, and more likely to undergo scheduled procedures, as opposed to urgent interventions when a condition has worsened. Insured individuals are, on average, also responsible for a lower percentage of the total cost. Because insured individuals still remain responsible for a proportion of the costs, insurance coverage on its own does not eliminate the risk of CSE [7,8]. In the United States, medical expenditures are the most common cause of bankruptcy [9].

The Green Family Foundation Neighborhood Health Education Learning Program (NeighborhoodHELP)

The Green Family Foundation Neighborhood Health Education Learning Program (NeighborhoodHELP) is an interprofessional medical and social service program serving underserved communities in Miami-Dade County. The program places individual medical students with interprofessional teams consisting of nursing, social work, physician assistant students, and outreach workers. The teams of medical and interprofessional students and a supervising faculty member collaborate to address the social determinants of health for each household assigned to them from designated low-income and uninsured communities [10,11]. Over the course of 12 years, the program has provided over 14,000 home visits and served more than 3,400 patients, with approximately 1,950 patients currently being served [12].

As part of its background data infrastructure, NeighborhoodHELP gathers contact information, basic demographic information, and longitudinal tracking of social needs and social risk factors. Although NeighborhoodHELP does not collect information on income and expenses at the household level, it does make use of household addresses and block-group level data to access publicly available indicators of social deprivation, like the Area Deprivation Index (ADI). Such indicators assist in the risk assessment of households and can alert providers of the potential need for resources [13]. Originally extrapolated from long-form census data by the US Department of Health and Human Services, the ADI is a composite, weighted score of 17 factors including employment status, educational achievement, housing stability, and poverty. Through a project called “Neighborhood Atlas,” a team at the University of Wisconsin-Madison School of Medicine and Public Health subsequently modified the ADI score and created a website capable of generating an ADI score from a household address, narrowed down to the block group level [14]. ADI scores range from one to 100, with higher numbers representing higher levels of social deprivation. The ADI has been used as a tool to inform other area-based efforts to improve population health [15,16]. NeighborhoodHELP does not, however, routinely calculate the risk of CHE or CSE among its households.

Estimating the risk of CSE among NeighborhoodHELP households

This modeling study aims to answer the question: How many households in NeighborhoodHELP are potentially at risk of CSE if they were to receive unexpected surgical care? By understanding the risk of CSE in this population, the study can help inform interventions to mitigate financial catastrophe associated with surgical care. Our team also asked two subsidiary questions: Is the ADI score associated with the calculated risk of CSE? If so, could the ADI score be used as a proxy indicator for the risk of CSE in at-risk populations?

The study is significant because it assesses the risk of CSE in a population vulnerable to financial catastrophe due to medical expenditures. The findings of this study can help inform interventions to mitigate the financial burden of surgical care in underserved communities, including the use of readily available place-based indicators like the ADI. The study's results can also inform policy discussions related to access to surgical care for uninsured and underinsured households in the United States.

## Materials and methods

Calculating risk of CSE

The aim of this study was to determine the risk of CSE among uninsured households enrolled in NeighborhoodHELP. To achieve this aim, we used established methodology from the World Health Organization (WHO) to calculate the risk of CSE for surgical procedures in this population [5,17]. All analyses throughout the study were conducted at the household level.

We obtained initial data on all 851 households enrolled in NeighborhoodHELP as of February 28, 2020. To estimate missing variables, including annual income, rent, and food expenditures, we used publicly available data from the US Census and the American Community Survey (ACS), referring to calendar year 2018 [18]. We focused on the risk of CSE for cholecystectomy, one of the most common surgical procedures in the US, and one that can occur unexpectedly. The Agency for Healthcare Research and Quality (AHRQ) periodically provides updates on the most common procedures done in the US. In 2018, the most recent year for which AHRQ has published a brief report, there were 335,200 cholecystectomies reported, the seventh most commonly performed procedure in hospital operating rooms [19].

To determine the risk of CSE, we used the WHO methodology, which defines a catastrophic expenditure as when the out-of-pocket (OOP) burden times the cost of a healthcare event exceeds a threshold for non-subsistence household expenditures. In order to determine these expenditures and the relevant income threshold, we relied upon publicly available data from the ACS at the census block group level. The ACS provides median values for rent and estimates of annual food expenditures. It also provides estimates of income for each census block, along with margins of error. For the analysis, we randomly selected income values within one standard deviation of the reported value, adjusted for household size.

In alignment with the WHO methodology, we set the threshold for CSEs at 40% of annual non-subsistence expenditure [20]. We created a dichotomous variable in which catastrophic expenditure occurs if the OOP payment for surgery exceeds 40% of what a household can pay [5,17,20].

Effects of varying rates of insurance coverage on risk of CSE

To assess how different rates of insurance coverage affect the risk of CSE, we adjusted the model according to different assumptions of the OOP costs. These different assumptions about insurance coverage rates, based on available literature, allow for modeling how the risk of CSE would change in different scenarios. In the WHO methodology for CHE, CSE is a subset of CHE, with CSE including the surgery-specific costs plus the surgery-related hospitalization costs. In other words, the expenditure used to estimate CSE would be captured in the global fee for a procedure and its related hospitalization costs.

To our knowledge, there are no published estimates of proportional costs for global surgery fees in the US by insurance status, but there are estimates of expected OOP costs for hospitalizations in general, regardless of the condition. Specifically, we considered three different rates of expected OOP costs asked of patients: 6.5%, 38%, and 100%. These percentages come from an extensive report conducted by the Institute of Medicine (IOM) (now called the National Academy of Medicine) in 2003, entitled Hidden Costs, Values Lost: Uninsurance in America. The IOM report examined nationwide data and contrasted the costs to finances and to overall health of being insured versus uninsured. 6.5% represents the average percentage costs of the total for a hospitalization for insured patients in the US; 38% represents the average percentage costs of the total for a hospitalization for uninsured patients; 100% indicates the total cost for the hospitalization [21]. Subsequent studies have found very similar percentages when it comes to the OOP costs incurred by the uninsured. A follow-up report by the IOM in 2009 calculated the total cost of health care services to the uninsured in 2008 as $86 billion, with the uninsured paying $30 billion, or 34.9%, of the costs out of pocket [22]. A more recent report, compiled by the Kaiser Family Foundation in 2023, estimates that the uninsured pay almost 40% of the costs for healthcare out of pocket [23]. Seeing this consistency, the investigators used the original IOM estimates for the models presented in this study.

To calculate the OOP costs for a cholecystectomy, we used cost data from the AHRQ for the State of Florida [24]. We assumed that uninsured patients would pay either 100% of the global fee for the procedure or negotiate down to an average of 38% of the costs, whereas insured patients would pay only their deductible and co-insurance amounts, on average, about 6.5% of the total costs. We used the AHRQ data to estimate the average cost of the procedures, the average length of stay in the hospital, and the average number of days of post-operative care.

Assessing for correlations with Area Deprivation Index scores

As mentioned above, the ADI is an analytical technique for measuring and quantifying the degree of socioeconomic disadvantage within a designated geographic area. For this study, we utilized 2018 ADI scores at the block group level, directly downloaded from the Neighborhood Atlas at the University of Wisconsin Applied Population Lab [14].

For the households enrolled in NeighborhoodHELP (851 at the time of this study), we maintain household-specific information, including the addresses, in a secure, HIPAA-compliant database. We geocoded those addresses at the block group level for the analysis, using ArcGIS 10.1 (Esri, Redlands, CA). After geocoding the household addresses and thus having their associated block group levels, we then obtained the ADI scores for those block groups from the Neighborhood Atlas database [14].

Once we calculated the risk of CSE for cholecystectomy, we compared these results to existing data on the ADI scores for the corresponding households. For each of the different scenarios proposed, assuming insurance or lack of insurance for the households, we first computed Phi-K correlation coefficients to capture any non-linear dependencies between variables. We then performed a logistic regression, looking for associations between the risk of CSE and the ADI score.

All calculations were performed using the R programming language, version 4.2.1 (R Foundation for Statistical Computing, Vienna, Austria). We used descriptive statistics to summarize the results and provide confidence intervals around our estimates.

Institutional Review Board approval

We obtained Institutional Review Board (IRB) approval for this study from Florida International University (FIU), Protocol # IRB-20-0351. Because the research was retrospective and involved only secondary data analysis, a waiver of informed consent was obtained. The researchers immediately de-identified the personal health information - physical addresses - once the Census Tract and Block Group codes were obtained, in order to protect patient privacy. 

Ethics statement

As indicated above, the study was approved, with an accompanying waiver of consent, by the FIU IRB. The FIU IRB was established under federal regulations for the protection of human subjects in research (45 CFR 46), with an express purpose to help protect the rights and welfare of human participants in research. The study was performed in accordance with the ethical standards as laid down in the 1964 Declaration of Helsinki and its later amendments.

## Results

Of the 851 households enrolled in NeighborhoodHELP, the 758 households for which we had adequate information to perform a calculation based on the WHO methodology were included in the study. The 758 households included in the study had a median non-subsistence expenditure of $27,043.50, with an interquartile range (IQR) of $22,091.75, the difference between the 75th percentile ($38,955.75) and the 25th percentile ($16,864.00). The median household size was 2 (IQR = 3; 75th percentile = 4; 25th percentile = 1), and the median ADI score was 59 (IQR = 29; 75th percentile = 75; 25th percentile = 46). Of the households, 314 (41.42%) fell below federal poverty standards (Table 1).

**Table 1:** Socioeconomic profile of study population: summary statistics (n = 758 households) ADI: Area Deprivation Index

	Non-subsistence expenditure (US dollars)	Household size	ADI score
Min	-1,759	1	1
25th percentile	16,864	1	46
Median (50th percentile)	27,044	2	59
Mean	30,171	2.8	59.4
75th percentile	38,956	4	75
Max	150,758	15	100

For the State of Florida in 2018, the average cost for a cholecystectomy was $17,875. Using this cost and assuming a 100% payment burden, 88.92% of households were at risk of CSE if required to pay for a cholecystectomy. However, assuming a 38% payment burden, the percentage of households at risk decreased to 43.90%. Finally, assuming a 6.5% payment burden, the percentage of households at risk decreased further to 7.70% (Table 2).

**Table 2:** Modeled risks of catastrophic surgical expenditure for study population (n = 758 households) CSE: catastrophic surgical expenditures

Surgical procedure	Average cost (US dollars) for procedure in Florida (2018)	Number and percentage of households at risk for CSE					
		Assumption 1: Household responsible for 100% of cost		Assumption 2: Household responsible for 38% of cost		Assumption 3: Household responsible for 6.5% of cost	
		Number at risk	% at risk	Number at risk	% at risk	Number at risk	% at risk
Cholecystectomy	17,875	674	88.92%	333	43.90%	58	7.70%

These CSE results were then analyzed in comparison with the known ADI scores for all of our households. We calculated Phi-K correlations to identify non-linear relationships between ADI scores and risk of CSE. We also performed three simple logistic regressions, looking for associations between the dichotomous (yes/no) risk of CSE and the numerical ADI scores, from one to 100. Using these pre-calculated variables: cholecystectomy in the uninsured (assuming 100% of the cost); cholecystectomy in the uninsured (assuming 38% of the cost); and cholecystectomy in the insured (assuming 6.5% of the cost), we performed logistic regression. 

Phi-K correlations between ADI and CSE risk were moderate when patients were responsible for 100% of costs and 38% of costs (0.64 and 0.52, respectively). For all regressions, the ADI scores showed associations with the risk of CSE, and these associations were statistically significant (at p < 0.05) for all models involving the uninsured. For the uninsured, each point increase in the ADI score was associated with an odds ratio of approximately 1.03 to 1.07 for an increased risk of CSE (Table 3).

**Table 3:** Correlations and logistic regression models, assessing for correlations and associations between risk of CSE and ADI scores ADI: Area Deprivation Index, CSE: catastrophic surgical expenditures

Model	Phi-K correlation	Confidence interval		Odds ratio	p-value	Statistical significance
Cholecystectomy						
Model 1: Cholecystectomy - uninsured, responsible for 100% of cost	0.641475	0.05812454	0.08879628	1.0755230	<2e-16	Statistically significant
Model 2: Cholecystectomy - uninsured, responsible for 38% of cost	0.517675	0.02951162	0.04570532	1.03814879	<2e-16	Statistically significant
Model 3: Cholecystectomy - insured, responsible for 6.5% of cost	0.230580	-9.141173e-05	0.02614851	1.01294249	0.0542	Not statistically significant

**Figure 1:**
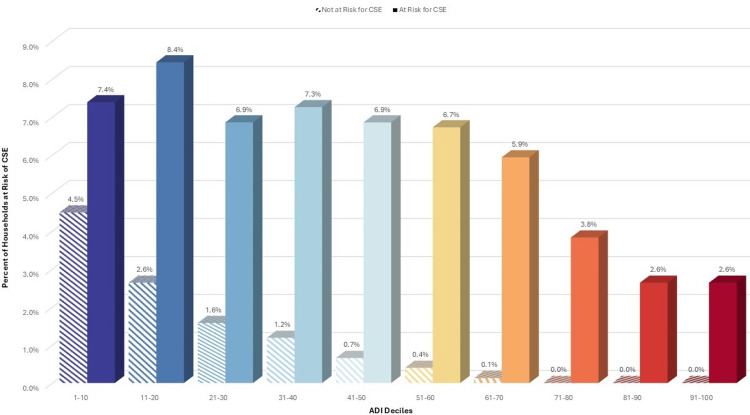
Risk of CSE for cholecystectomy among uninsured responsible for 100% costs, by ADI decile ADI: Area Deprivation Index, CSE: catastrophic surgical expenditures

**Figure 2:**
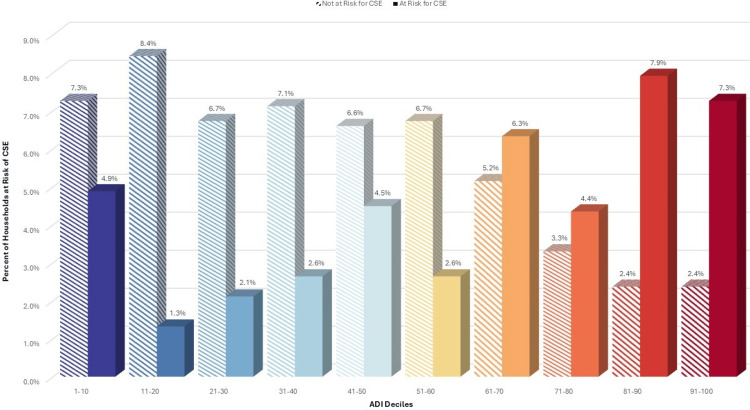
Risk of CSE for cholecystectomy among uninsured responsible for 38% costs, by ADI decile ADI: Area Deprivation Index, CSE: catastrophic surgical expenditures

**Figure 3:**
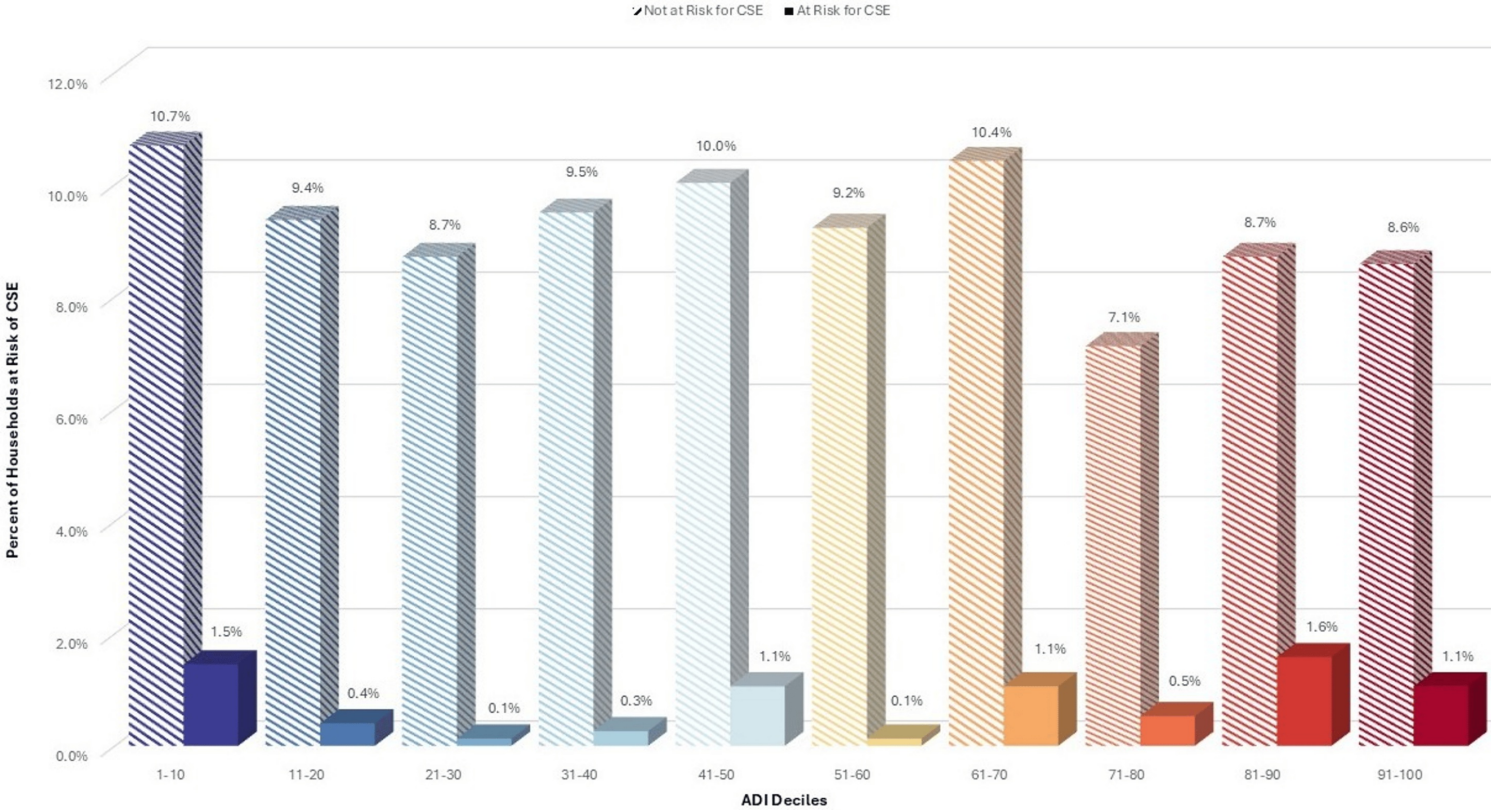
Risk of CSE for cholecystectomy among insured responsible for 6.5% costs, by ADI decile ADI: Area Deprivation Index, CSE: catastrophic surgical expenditures

When grouping ADI scores by deciles, the associations with the risk of CSE gradually increase with higher deciles, especially among the uninsured, who are responsible for 38% of the total cost (Figures 1-3).

## Discussion

These results demonstrate the high level of financial vulnerability among the households in our study. With an estimated median non-subsistence expenditure of $27,043.50, many of these households may struggle to afford even routine healthcare expenses, let alone emergency surgeries. The fact that over 40% of households fell below federal poverty standards underscores the significant financial challenges faced by these families.

The risk of CSE for uninsured patients is particularly striking. Even assuming a reduced payment burden of 38%, over 40% of households in our study would be at risk for CSE. This risk highlights the potential devastating impact that a medical emergency can have on the financial well-being of these households. In contrast, for insured patients, the risk of CSE is much lower. Assuming average costs for insured patients for a cholecystectomy in 2018, only 7-8% of households would be at risk for CSE.

Recognizing the high risks of CSE among underserved households in our program, we will now examine some of the implications of these findings, some possible strategies for mitigating such risks, and some of the limitations of our study. In addition, we will explore the potential of using this model as a screening tool to identify high-risk patients in similar populations and how the ADI score might be used as a proxy indicator of CSE. Finally, we will offer suggestions for future research and interventions and for broader healthcare policy.

Upstream, midstream, and downstream implications

One way to examine the impacts of social factors, such as financial instability, on health and healthcare is to explore the implications at what have come to be known as upstream, midstream, and downstream points of intervention. In broad strokes, acting upstream involves community-level interventions to improve local conditions, and midstream approaches focus on addressing an individual’s social needs. Downstream approaches focus on medical interventions and on treating illnesses and sequelae of illnesses [25,26].

The striking findings of our study involving an underserved population with high rates of uninsurance suggest that providing health insurance could have profound impacts on preventing the risks of CSE. At the upstream level, laws, policies, and regulations that expand insurance access would reduce the risks of CSE among underserved households in our program. In states such as Florida that have not expanded Medicaid, one of only 10 states not to have done so [27], such expansion could be an effective longer-term solution toward preventing CSE. Other programs that provide specific coverage for individuals facing CHE/CSE or structured insurance coverage within pre-paid financing models could also be enacted [28,29].

At the midstream level, addressing individuals' social needs and providing financial assistance are possible tactics. Including screening questions or other available indices to identify individuals at risk and using data to refer to others who can assist are key tactics that can be used. Partnering with social services, community health, and other community organizations can also be an effective strategy toward finding available resources for at-risk households. Law professionals can also be involved in advocating for debt forgiveness and for the provision of financial assistance to underserved households [25].

At the downstream level, providing patient-centered clinical care and tailored medical interventions that take costs into account are recommended. Such care can involve collaborating with other medical and nursing professionals to provide affordable medical interventions to patients experiencing potential CSE [26].

Potential screening tool

The potential of using this model as a screening tool deserves further exploration. Including screening questions or other available indices to identify individuals at risk of CSE could be an effective strategy. This approach could help identify at-risk households and guide decision-makers and clinicians [30]. By screening ahead of time and tailoring interventions to particular patients and households, we can reduce the risks of CSE among underserved households.

Currently, some hospitals and health systems have mechanisms for identifying patients at risk of being unable to afford their payments, and they direct these individuals toward charity care providers and payment plans. Once a patient is in debt, some hospitals also offer debt forgiveness programs if patients meet certain hospital-specific criteria. Standardizing this type of screening and support for patients, using recognized criteria, may help prevent the more devastating downstream financial effects of medical treatment. 

Area Deprivation Index

Because calculating the risk of CSE can be cumbersome and requires gathering sensitive household information like income and costs for rent and food, it is worth exploring other proxy indicators that correlate with CSE but are easier for policy-makers, administrators, and clinicians to determine. Our models demonstrating a strong association between higher ADI scores and the risk of CSE for the uninsured could point toward one way to identify those at higher risk of CSE among different population groups. With every one-point increase in ADI score increasing the odds ratio of CSE by about 1.05, hospital administrators, clinic managers, and clinicians can use this estimate to determine ADI scores for their clients and flag those most at risk for CHE. Our population points toward ADI scores of 61 and higher as being at particular risk. Further research could help identify thresholds of ADI scores that indicate those most in need of support and target interventions accordingly [16,31].

Independent components

Another approach toward identifying proxy indicators of the risk of CSE would involve exploring the independent components that contribute to the risks of CSE among underserved households [32,33]. If, for example, a simpler indicator such as household income in relation to the federal poverty level is found to have a strong association with CSE, such a factor could be used as a prompt for noting risk and/or interventions. Further research could evaluate factors such as income, education, and access to healthcare as independent components that contribute to the risks of CSE among underserved households.

Degree of catastrophic expenditure burden

It is crucial to identify the level of financial burden patients are experiencing as a “catastrophic” expenditure. The expected cost burden on a particular household, should there be an unexpected health emergency, would help us understand the severity of the problem and tailor interventions accordingly. The WHO method theorizes CSE as a dichotomous variable, but it is important to identify whether patients are experiencing excess expenditure over what they can afford at the $100 versus the $10,000 level. Similarly, there may be patients who fall just below the threshold for a "catastrophic" expenditure for whom an emergent surgery would still be a significant financial strain. Developing continuous models that estimate variable degrees of financial strain may prove more fruitful for health care providers and systems. 

Study limitations

Our study has several limitations that should be considered when interpreting the results. First, our sample was drawn from a single geographic area and may not be representative of the broader population. Second, we relied on census data and a randomization protocol established by the WHO for estimating individual household income and expenditure; such calculations meet expected population health research guidelines but may miss the subtleties of individual household realities. According to internal program data at the time of household enrollment, by self-report, 80.4% of NeighborhoodHELP household members are uninsured. This self-report data suggests that our estimates of the risk of CSE may be overestimated. Even so, the risks would be significant, suggesting that at least 71.5% of our households would be at risk of CSE if expected to pay 100% of the costs of a cholecystectomy, and 35.3% would be at risk if asked to pay 38%. This level of risk matches that found in much of the developing world [5]. Finally, our analysis focused on only one type of surgery and may not be generalizable to other types of medical emergencies.

Future directions

The high risk of CSE for uninsured patients highlights the need for policies that expand access to health insurance, particularly for low-income families. Our models also underscore the importance of efforts to reduce healthcare costs and increase transparency around healthcare pricing, in order to reduce the financial strain for families facing medical emergencies.

Finally, the study points toward areas of further research to help clinicians and health systems efficiently and accurately identify households at risk and to target interventions to prevent long-term financial fallout from medical care.

## Conclusions

Our study provides important insights into the financial vulnerability of households in our sample. With over 40% of households falling below federal poverty standards, the high risk of CSE for uninsured patients facing emergency surgeries is not unexpected. These models, nevertheless, reveal patterns among the households at particular risk, showcase the dramatic effects of differing levels of OOP costs and insurance coverage on these risks, and suggest multiple avenues for intervention. They further underscore the need for clinically relevant and easy-to-calculate ways to identify high-risk households and suggest the utility of place-based indicators of social deprivation, like the ADI, in that vein.
